# CMV Seropositive Status Increases Heparanase SNPs Regulatory Activity, Risk of Acute GVHD and Yield of CD34^+^ Cell Mobilization

**DOI:** 10.3390/cells10123489

**Published:** 2021-12-10

**Authors:** Olga Ostrovsky, Katia Beider, Yan Morgulis, Nira Bloom, Angel Cid-Arregui, Avichai Shimoni, Israel Vlodavsky, Arnon Nagler

**Affiliations:** 1 Chaim Sheba Medical Center, Department of Hematology and Bone Marrow Transplantation, Tel-Hashomer, Ramat Gan 5266202, Israel; katiabeider@gmail.com (K.B.); Yan.Morgulis@sheba.health.gov.il (Y.M.); Nira.Bloom@sheba.health.gov.il (N.B.); avichai.shimoni@sheba.health.gov.il (A.S.); Arnon.Nagler@sheba.health.gov.il (A.N.); 2 German Cancer Research Center, D-69120 Heidelberg, Germany; a.cid@dkfz-heidelberg.de; 3 Technion Integrated Cancer Center, Rappaport Faculty of Medicine, Technion, Haifa 3525433, Israel; vlodavsk@mail.huji.ac.il

**Keywords:** HPSE gene, CMV, SNPs, enhancer, insulator, allogeneic HSCT, acute GVHD, G-CSF-mobilization

## Abstract

Heparanase is an endo-β-glucuronidase that is best known for its pro-cancerous effects but is also implicated in the pathogenesis of various viruses. Activation of heparanase is a common strategy to increase viral spread and trigger the subsequent inflammatory cascade. Using a Single Nucleotide Polymorphisms (SNP)-associated approach we identified enhancer and insulator regions that regulate HPSE expression. Although a role for heparanase in viral infection has been noticed, the impact of HPSE functional SNPs has not been determined. We investigated the effect of cytomegalovirus (CMV) serostatus on the involvement of HPSE enhancer and insulator functional SNPs in the risk of acute graft versus host disease (GVHD) and granulocyte-colony stimulating factor related CD34^+^ mobilization. A significant correlation between the C alleles of insulator rs4364254 and rs4426765 and CMV seropositivity was found in healthy donors and patients with hematological malignancies. The risk of developing acute GVHD after hematopoietic stem cell transplantation was identified only in CMV-seropositive patients. A significant correlation between the enhancer rs4693608 and insulator rs28649799 and CD34^+^ cell mobilization was demonstrated in the CMV-seropositive donors. It is thus conceivable that latent CMV infection modulates heparanase regulatory regions and enhances the effect of functional SNPs on heparanase function in normal and pathological processes.

## 1. Introduction

Heparanase (HPSE) is an endo-β-glucuronidase that specifically cleaves the saccharide chains of heparan sulfate (HS) proteoglycans (HSPG), key components of the cell surface, basement membrane and extracellular matrix (ECM). Cleavage of HS by active heparanase results in impairment of the basement membrane and ECM integrity and the release of HS-bound chemokines, cytokines and growth-promoting factors [1].

Heparan sulfate and heparanase are implicated in the pathogenesis of various unrelated viruses [2,3]. HS has long been known to serve as an attachment receptor for a large variety of human viruses, including herpes simplex virus (HSV), dengue virus (DENV), respiratory syncytial virus (RSV), varicella-zoster virus (VZV), hepatitis C virus (HCV), human immunodeficiency virus (HIV), human papillomavirus (HPV), Epstein–Barr virus (EBV), Vaccinia virus (VACV) and others [4,5,6,7,8,9,10,11,12,13,14]. Furthermore, recent studies have shown that heparanase plays a role in regulating the lifecycle and/or dissemination of pathogenic viruses [12,13,14,15,16,17]. Of particular importance is the binding of the SARS-CoV-2 spike protein to HS prior to its interaction with the angiotensin-converting enzyme-2 (ACE2) receptor [18]. Notably, increased heparanase activity and HS levels were found in the plasma of COVID-19 patients, associating with the severity of the disease [19]. It appears that up-regulation and activation of heparanase increase the spread of various viruses and likely trigger the subsequent inflammatory cascade [12,14].

In previous studies, we identified two regulatory regions of the HPSE gene, a strong enhancer, located in intron 2 [20], and a 200 bp insulator mapped in intron 9 [21]. The number of functional SNPs in both regulatory regions and their interactions were characterized [21]. The enhancer rs4693608 SNP has a major impact on HPSE gene expression and the risk of acute graft-versus-host disease (GVHD) after hematopoietic stem cell transplantation (HSCT). The C alleles of insulator SNPs rs4364254 and rs4426765 modify the activity of the HPSE gene enhancer resulting in both altered heparanase expression and increased risk of developing acute GVHD [21]. In addition, the enhancer rs4363084 SNP and insulator rs28649799 SNP were shown to be associated with the yield of granulocyte-colony stimulating factor (G-CSF) mediated CD34^+^ cell mobilization in normal donors. Moreover, rs4426765 correlates with HPSE gene expression in activated mononuclear cells (MNCs), and with CD3 cell number and lymphocyte counts in response to G-CSF-induced cell mobilization [21].

Although a role for heparanase in viral infection has been identified, the impact of HPSE gene functional SNPs has not been determined. Cytomegalovirus (CMV) is an ancient herpes virus that co-evolved with its host over millions of years and persists as a sub-clinical and lifelong infection. At a late stage of primary infection, viral gene expression is halted in some cells, resulting in episomal viral genomes that remain in the nucleus [22]. This state of latency appears to be restricted to bone-marrow CD34^+^ cells and CD33^+^ myeloid progenitor cells. These cells retain a latent episomal viral genome and differentiate into peripheral blood monocytes and myeloid dendritic cells, which are the predominant site of latency in healthy subjects [23].

The present study analyzed the correlation between functional heparanase SNPs and CMV seropositivity. We assessed the involvement of the previously discovered enhancer and insulator SNPs [21] in the risk of acute GVHD and the yield of G-CSF mediated CD34^+^ mobilization in CMV seropositive versus seronegative individuals. Our results revealed a significant correlation between the C alleles of insulator rs4364254 and rs4426765 SNPs and susceptibility to CMV infection in healthy donors and patients with acute myeloid leukemia (AML), acute lymphoblastic leukemia (ALL), and myelodysplastic syndrome (MDS). Correlation analysis between functional HPSE SNPs and the risk of developing acute GVHD after HSCT revealed a significant association only in CMV-seropositive patients, as opposed to CMV-seronegative patients. The study of functional HPSE SNPs and G-CSF-associated cell mobilization revealed a significant correlation between the enhancer rs4693608 and insulator rs28649799 SNPs and the extent of CD34^+^ mobilization, only in the CMV-seropositive donor group.

Our results indicate that latent CMV infection modulates the regulatory regions of HPSE and thus may enhance the effect of functional SNPs on the involvement of heparanase in normal and pathological processes.

## 2. Materials and Methods

### 2.1. Study Population

The retrospective study included 523 patients (303 males and 219 females) with the following hematologic malignancies: acute myeloid leukemia (AML), acute lymphoblastic leukemia (ALL), chronic myeloid leukemia (CML), chronic lymphocytic leukemia (CLL), Hodgkin’s lymphoma (HL), myelodysplastic syndrome (MDS), myelofibrosis (MF), multiple myeloma (MM), Non-Hodgkin’s lymphoma (NHL), and severe aplastic anemia (SAA). The study was approved by the Ethics Committee of the Sheba Medical Center and the Israeli Ministry of Health (#4247). All subjects gave their written informed consent. Two hundred and sixty-one patients received grafts from human leukocyte antigen (HLA)-identical siblings, and 262 patients were transplanted from unrelated donors. The median age was 52 years (range 17 to 79 years). Conditioning regimens before HSCT, administration of G-CSF, and anti-GVHD prophylaxis were in accordance with the previously described departmental routine [21,24,25].

CMV serostatus were assessed using the ARCHITECT CMV IgM and IgG assays (Abbott, Sligo, Ireland). The presence or absence of anti-CMV IgM and IgG in the plasma was determined by comparing the chemiluminescent signal of the reaction to the cutoff signal determined by calibration for IgM or IgG. If the chemiluminescent signal in the plasma was greater than or equal to the cutoff signal, the plasma was considered reactive for anti-CMV IgM or IgG, respectively. The cutoff signal for IgM is <0.85 Index Value and for IgG it is <6.0 AU/mL.

Four hundred and eighty-two healthy donors were included in the study to assess the involvement of functional HPSE SNPs in the risk of CMV infection. The effect of the HPSE SNPs on G-CSF-mediated peripheral blood stem cell mobilization was analyzed in 215 consenting healthy stem cell normal donors after the first apheresis. The stem cell donors received G-CSF for 5 days, as previously described [21,26].

### 2.2. SNPs Analysis

The genotypes of functional HPSE gene SNPs were performed as previously described [20,21]. In brief, the genotypes of rs4693084 and rs4364254 SNPs were detected by allele-specific amplification. Polymerase chain reaction (PCR) fragments were amplified from genomic DNA (Wizard^®^ Genomic DNA Purification kit, Promega, Madison, WI, USA) using the forward and reverse primers. Conditions of the PCR reactions were previously published [20]. Real-Time SNP assay was applied for rs4693608, rs4426765, and rs28649799 SNPs genotyping using custom-specific primers and probes (Bio Search Technologies, Novato, CA, USA). The PCR reactions were performed using an ABI PRISM 7700 sequence detector (Applied Biosystems, Warrington, UK) according to the manufacturer’s instructions (Quanta, Gaithersburg, MD, USA).

### 2.3. Statistical Analysis

All analyzed individuals were divided into CMV-seropositive and CMV-seronegative and statistical analysis was performed for each group separately. Genotype and allele frequencies of the SNPs were calculated by direct counting. The χ^2^ test was used to assess categorical variables, and the Mann–Whitney U test was applied for continuous variables. Statistical analysis was performed for grades II-IV acute GVHD. The cumulative incidence of acute GVHD was calculated for both enhancer and insulator HPSE SNPs, as previously described [21]. Time to clinical event was measured from the date of HSCT. Relapse was considered a competing risk. The role of the discrepancy between recipients and donors in functional HPSE SNPs was assessed for CMV-seropositive and CMV-seronegative groups. To evaluate the significance, we applied the Gray’s test. Gray’s test compares subgroups while considering competing risks using the sub-distribution hazards. G-CSF-mediated peripheral blood stem cell mobilization was analyzed using the Mann–Whitney U test in accordance with a previously published approach [21]. A *p*-value ≤ 0.05 was considered statistically significant and was presented after approximation with corrections. The calculations were performed using the NCSS software 2021 (NCSS, Kaysville, UT, USA).

## 3. Results

### 3.1. Correlation between Enhancer and Insulator HPSE SNPs and CMV Seropositivity in Healthy Donors and Patients with Hematological Malignancies

The genotype and alleles frequencies of two enhancer SNPs (rs4693608 and rs4693084) and three insulator SNPs (rs4426765, rs28649799 and rs4364254) were calculated for 329 CMV-seropositive and 153 CMV-seronegative healthy donors. SNPs analysis revealed a significant correlation between rs4364254 SNP and CMV seropositivity (Table 1). The frequency of the CC genotype and the C allele was higher in CMV-seropositive individuals compared to CMV-seronegative people (13.5% versus 6.1%, *p* = 0.041 for genotype CC, and 36% versus 27.5%, *p* = 0.015 for allele C, respectively, Table 1).

Analyzing heterozygous individuals for predominant SNPs may help to better understand the interaction between enhancer and insulator SNPs in the HPSE gene [21]. Calculation of all possible genotypes of rs4693608, rs4364254 and rs4426765 SNPs disclosed that not only rs4364254 but also rs4426765 have an effect on the susceptibility to CMV infection (Table 2). The frequency of AG-TC-AA genotype was similar among CMV-seropositive and seronegative individuals. However, the frequency of the AG-TC-AC genotype in CMV-seropositive donors was higher compared to the frequency of this genotype in CMV-seronegative individuals (15.9% versus 10.7%) (Table 2). The overall frequency of other genotypes (AG-TC-CC, AG-CC-AC, AG-CC-CC, and AG-CC-AA) was higher in CMV-seropositive compared to CMV-seronegative donors (8.5% versus 1.6%). To assess the statistical difference between rs4693608, rs4364254 and rs4426765 SNP genotypes, we categorized all variants into three groups A, B, and C (Table 2). In group B, the frequency of CMV-seropositive genotypes was twice as high as the frequency of CMV-seronegative genotype (24.4% versus 12.4%, *p* = 0.025), while in groups A and C the same genotype variability was observed. A definite effect of the C alleles of rs4364254 and rs4426765 SNPs on the susceptibility to CMV infection in healthy individuals is noticeable (Table 2). However, the frequencies of genotypes AG-TC-CC, AG-CC-AC, AG-CC-CC, and AG-CC-AA are relatively low in the general population. Therefore, additional future analysis of large population samples is required.

Genotype and allele frequency analysis of 5 HPSE gene SNPs (rs4693608, rs4693084, rs4426765, rs28649799 and rs4364254) in all 523 patients with hematological malignancies did not reveal a correlation with CMV seropositivity. However, a significant correlation between two insulator SNPs (rs4364254 and rs4426765) and the susceptibility to CMV infection was identified in patients with AML (Table 3). In CMV-seropositive patients, the frequency of the TT rs4364254 genotype was low compared to CMV-seronegative individuals, while the frequency of the TC genotype was significantly higher than in the CMV-seronegative patient group (*p* = 0.002). The C alleles frequencies of rs4364254 and rs4426765 SNPs were significantly higher in CMV-seropositive AML patients compared to CMV-seronegative patients (*p* = 0.006 for rs4364254 and *p* = 0.024 for rs4426765, respectively) (Table 3).

Since the small number of CMV-seronegative patients with other hematological malignancies did not allow for correlation analysis, we compared them with CMV-seropositive and CMV-seronegative healthy donors (Table 4). Involvement of insulator SNPs in susceptibility to CMV infection was found in acute lymphoblastic leukemia (ALL), myelodysplastic syndrome (MDS), and multiple myeloma (MM). The most significant correlation was observed between rs4426765 SNP and CMV-seropositive ALL patients. Significant differences were detected compared to both CMV-seropositive and CMV-seronegative controls (*p* = 0.034 and *p* = 0.008, respectively) (Table 4). These results suggest an important role for the HPSE intron 9 insulator in susceptibility to CMV infection.

### 3.2. Impact of CMV Infection on the Involvement of Heparanase SNPs in the Risk of Acute GVHD

Our previous studies have shown that among several functional HPSE SNPs, the enhancer rs4693608 SNP has a major effect on the risk of acute GVHD post-HSCT [21,24,25]. The C alleles of insulator SNPs rs4364254 and rs4426765 modulate the activity of the HPSE gene enhancer and thereby increase the risk of acute GVHD [21]. Given the interaction of enhancer and insulator SNPs, all individuals were divided into three groups N-HR, N-MR, and N-LR with high, medium and low risks of developing acute GVHD. In addition, disparities between recipient and donor in SNP combinations of the HPSE gene significantly increased the likelihood of acute GVHD morbidity [21]. As a result, all recipient-donor pairs were divided into three groups (D1, D1 and D3) in accordance with the potential risk of developing acute GVHD [21,24]. The distribution of all individuals into groups is illustrated in Table 5.

We aimed at assessing the CMV seropositivity component in heparanase SNP-associated risk for acute GVHD after HSCT. The risk of acute GVHD was investigated in 462 CMV-seropositive and 61 CMV-seronegative patients as well as 329 seropositive and 153 seronegative donors. Univariate analysis of the cumulative incidence of clinically significant acute GVHD (grades II-IV) relative to CMV status, patient enhancer rs4693608, patient enhancer-insulator SNP groups (N-HR, N-MR and N-LR), and discrepancy groups (D1, D2, D3) is presented in Table 5. Analysis of the cumulative incidence of acute GVHD on day 100 relative to CMV status revealed an increased risk of acute GVHD only in CMV-seropositive recipients as opposed to CMV-seronegative patients [39.7% (95% CI 35.3–44.7) versus 24.1% (95% CI 15.0–38.7), *p* = 0.03, Table 5]. No influence of CMV donor status was observed (Table 5).

Next, a univariate analysis of the cumulative incidence of clinically significant acute GVHD in correlation with the HPSE gene SNPs was performed separately in CMV-seropositive and CMV-seronegative groups. A significant impact of the HPSE gene SNPs was observed only in CMV-seropositive patients, but not in CMV-seronegative patients (Table 5). Best results were obtained in correlation with the patient’s HPSE enhancer-insulator groups. The cumulative incidence of acute GVHD was 51.8% (95% CI 44.2–60.6) in the N-HR group, 36.7% (95% CI 30.1–44.7) in the N-MR group, and 22.5% (CI 95% 15.0–33.8) in the N-LR group, respectively (*p* = 0.00022) (Table 5).

Our results further revealed that the effect of the discrepancy between recipients and donors in the HPSE gene SNPs was found only in the CMV seropositive patients. The cumulative incidence of acute GVHD was 58.9% (95% CI 47.4–73.3) in the D1 group, 41.1% (95% CI 35.0–48.3) in the D2 group, and 22.5% (CI 95% 15.0–33.8) in the D3 group (*p* = 0.00042) (Table 5). No impact of donor CMV status on the correlation between HPSE gene SNPs and the risk of acute GVHD was observed (Table 5).

### 3.3. Influence of CMV Serostatus on G-CSF-Mediated Peripheral Blood Stem Cell Mobilization

We recently reported the involvement of HPSE SNPs in G-CSF-mediated peripheral blood stem cell mobilization [21]. Enhancer rs4363084 and insulator rs28649799 SNPs were found to be associated with CD34^+^ cell mobilization yield. Moreover, SNP rs4426765 was found to correlate with CD3 cell numbers and lymphocyte counts in response to G-CSF-induced cell mobilization [21]. The effect of CMV-serostatus on heparanase-related G-CSF mobilization was investigated. The analysis included 215 donors (72 females and 143 males) and the median age was 45 (range, 19–73) years. One hundred and fifty-eight donors were CMV-seropositive (IgG—positive and IgM—negative) and 57 donors were CMV-seronegative (IgG—negative and IgM—negative). Association between the HPSE gene SNPs and the number of mobilized CD34^+^ cells after the first apheresis revealed a significant correlation between the enhancer rs4693608 SNP and the CD34^+^ cell numbers in CMV-seropositive donors (Table 6). The overall CD34^+^ yield, as well as the percentage of CD34^+^ cells, were higher in carriers of the AA genotype compared to possessors of the AG and GG genotypes (*p* = 0.022 and *p* = 0.007, respectively) (Table 6). No associations were found in CMV seronegative donors (Table 6). Similar results were observed in CMV-seropositive donors when the correlation between insulator rs28649799 SNP and G-CSF-induced CD34^+^ cell mobilization was analyzed. The level of CD34^+^ was higher in donors with genotype AG compared to donors with genotype AA (*p* = 0.031, Table 6).

Altogether, enhancer rs4693608 and insulator rs28649799 SNPs were associated with the process of G-CSF stem cell mobilization in CMV-seropositive donors. These two SNPs had opposite effects. The G allele of rs28649799, correlated with a high CD34^+^ cell number, had a low frequency and was found in partial linkage disequilibrium with the G allele of rs4693608, associated with a low CD34^+^ cell number. To clarify this complex association, we performed an additional correlation analysis. The analyzed group included rs28649799 AA donors, while individuals with the AG genotype, shown to correlate with a higher CD34^+^ mobilization yield, were excluded from the statistical analysis. Total, CMV-seropositive and CMV-seronegative donor groups were analyzed (Table 7). CMV-seropositive donors with genotype AA-AA exhibited a high level of CD34^+^ cell mobilization, while CMV-seropositive carriers of AA-AG and AA-GG genotypes disclosed medium and low levels of CD34^+^ cells (*p* = 0.021 for overall CD34^+^ yield; *p* = 0.003 for the percentage of CD34^+^) (Table 7). No correlation was found in the CMV-seronegative donors’ group (Table 7). We hypothesize that these enhancer and insulator SNPs have an enhancing effect in CMV-seropositive subjects. We had only three individuals with genotypes AA for rs4693608 and AG for rs28649799. The first was CMV-seronegative and showed a low yield of mobilized CD34^+^ cells (645.5 × 10^6^ for total CD34^+^ yield, 0.49 for CD34^+^ percentage), while the two other donors were CMV-seropositive and exhibited a very high yield of CD34^+^ cell mobilization (1535.1 × 10^6^ and 1035.3 × 10^6^ for total CD34^+^ yield; 1.21 and 1.03 for CD34^+^ percentage, respectively).

## 4. Discussion

Cytomegalovirus (CMV) is an opportunistic DNA virus that infects most adults worldwide. It is the largest and most complex of all human herpesviruses [27,28]. Initiation of human CMV requires initial interaction with cell surface heparan sulfate [29,30]. CMV is transmitted through all body fluids, including saliva and breast milk [28]. Cytomegalovirus exhibits a life-long latency and persistence. The viral genome remains in an inactive form predominantly in the population of CD34^+^ hematopoietic precursor cells located in the bone marrow. Latent CMV can be reactivated when the progenitor cells differentiate into macrophages or dendritic cells, resulting in spread of the virus into various cells and organs [31]. The proteins encoded by CMV affect adaptive immune responses, escape immune recognition, and thus elude its clearance from the host. While primary infection and reactivation of CMV infection rarely cause clinical symptoms in healthy individuals, the virus can lead to life-threatening illnesses in immunocompromised patients [28]. Herpesviruses in general are excellent regulators of the immune response and can survive successfully in an immune host [32]. Since herpesviruses affect most people, it is worth considering how herpesviruses influence the outcome of other co-infections, tumors, and grafts. Notably, herpesviruses can fine-tune the host’s immune system by modulating immune responses to make the host more resistant or susceptible to other disease conditions [32].

We report that the C alleles of HPSE rs4364254 and, to a lesser extent, rs4426765 insulator SNPs exhibit increased susceptibility to CMV infection in both healthy individuals and AML patients. These results indicate the relevance of the HPSE gene insulator for primary CMV infection.

Many research groups have demonstrated the high prevalence of CMV in various malignancies such as colon, breast, prostate, and hepatocellular cancers, neuroblastoma and brain tumors [33,34,35,36,37,38]. More than 90% of tumors are positive for CMV infection. The virus infection is confined to malignant and inflammatory cells and is not detected in adjacent normal cells [28]. The exact role of CMV as a cancer-associated agent in human tumors has not been elucidated and hence the virus is not included in the list of oncogenic viruses. However, CMV infection exhibits broad cellular tropism and is present in tumor epithelial cells, macrophages, endothelial and stromal cells [28]. Several studies have shown that the level of CMV infection in tumors negatively correlates with a positive outcome of the disease [33,39,40]. Treatment with antiviral therapy in cancer patients seropositive for CMV indicates an improved prognosis and potentially represents an effective anti-cancer strategy. Moreover, CMV gene products have been found to regulate multiple pro-tumorigenic cellular pathways [41].

SNP analysis of the HPSE gene in CMV-seropositive patients with hematological malignancies revealed a significant correlation between the incidence of ALL and insulator rs4426765 SNP. CTCF plays a universal and conserved role in the regulation of complex transcripts in the herpesvirus family [42]. Our previous study [21] identified a role of the CTCF-associated rs4426765 SNP in lymphocyte activation and function. The C to A substitution in rs4426765 disrupts the CTCF binding site. EMSA analysis in B- and T-ALL patient samples revealed that a large number of nuclear proteins bind to the insulator region containing the CTCF binding site [21]. The size of DNA/protein complexes decreased with a gradual distance from SNP rs4426765 to distal polymorphisms along the insulator nucleotide sequence [21]. The present results suggest that modulation of the HPSE gene insulator by CMV infection may contribute to the pathogenesis of ALL.

Acute GVHD and CMV replication are pathogenically related. Multiple studies show that acute GVHD and its treatment place patients at risk for CMV replication [43]. It was also demonstrated that patients with active CMV replication have an increased risk of developing acute GVHD [43]. In our previous studies [21,24,25], we found significant correlations between enhancer and insulator HPSE gene SNPs and the risk of acute GVHD post-HSCT. Moreover, the discrepancy between recipient and donor in HPSE SNPs contributes to the development of acute GVHD. In the present study, we assessed whether components of the patient’s and donor’s CMV serostatus may affect heparanase SNP-associated risk of acute GVHD after HSCT. We show that the previously found correlation of HPSE SNPs and the risk of acute GVHD was limited mainly to CMV-seropositive recipients. These results suggest that common mechanisms may underlie heparanase and CMV involvement in the development of acute GVHD. The risk of acute GVHD was high in patients that possessed a strong HPSE enhancer and a weak insulator (individuals in the N-HR group), while the donors had a strong HPSE insulator and weak enhancer (individuals in the N-LR group) or intermediate enhancer and insulator compared to the recipients (individuals in the N-MR group). The disparity in cell signaling can lead to hyperactivation of donor effector cells towards recipient tissues. Future studies will elucidate the role of latent CMV infection in the modulation of HPSE regulatory regions and explain the observed differences between recipients and donors.

Our study revealed that CMV serostatus correlates with CD34^+^ cell mobilization in HPSE gene SNP-dependent manner. Importantly, the amplifying effect of the enhancer rs4693608 and the insulator rs28649799 on the level of mobilized CD34^+^ cells was observed only in CMV-seropositive donors.

The involvement of heparanase in the viral life cycle was first discovered while studying the release of HSV-1 [44]. Subsequent studies have identified a role of heparanase in penetration of the virus into cells, increasing the severity of viral disease and facilitating viral egress from infected host cells [2,3]. The present study revealed possible involvement of the HPSE gene insulator in susceptibility to CMV infection and showed that CMV serostatus alters previously found correlations with the risk of acute GVHD and the yield of CD34^+^ cell mobilization. We propose that latent CMV may use the HPSE insulator and enhancer not only to better maintain the CMV latency but also to ensure efficient spread of the virus in the event of virus activation. It is conceivable that the CMV virus may promote high levels of heparanase either by increasing HPSE enhancer activity or by blocking HPSE insulator function

Virome plays an important role in cancer progression via mechanisms that involve inflammation, genotoxins, or oncogenes that alter the regulation and transcription of pro-tumorigenic human genes [45]. The vast majority of people carry herpesviruses as part of their virome causing no serious harm in healthy individuals [32]. Our results suggest that the presence of CMV leads to fine-tuning of the regulation of HPSE and likely other host genes in disease-prone situations. Detection of such mechanisms is crucial for the effective treatment of a variety of diseases ranging from acute and chronic inflammation to various cancers and autoimmune diseases.

## Figures and Tables

**Table 1:** Genotype and allele frequencies of the HPSE gene SNPs in CMV seropositive and seronegative healthy individuals.

SNP	Genotypes and Alleles	CMV-Seropositive		CMV-Seronegative		*p*-Value
		№	Incidence (%)	№	Incidence (%)	
rs4693608	AA	81	27.6	48	36.1	0.15
	AG	146	49.8	54	40.6	
	GG	66	22.5	31	23.3	
					
	A	308	52.6	150	56.4	0.3
	G	278	47.4	116	43.6	
rs4693084	GG	175	64.6	78	64.5	1
	GT	87	32.1	39	32.2	
	TT	9	3.3	4	3.3	
					
	G	437	80.6	195	80.6	0.99
	T	105	19.4	47	19.4	
rs4426765	AA	143	52.6	73	60.8	0.25
	AC	110	40.4	42	35	
	CC	19	7	5	4.2	
					
	A	396	72.8	188	78.3	0.1
	C	148	27.2	52	21.7	
rs28649799	AA	224	82	95	79.2	0.7
	AG	48	17.6	24	20	
	GG	1	0.4	1	0.8	
					
	A	496	90.8	214	89.2	0.46
	G	50	9.2	26	10.8	
rs4364254	TT	120	41.5	67	51.1	**0.041**
	TC	130	45	56	42.7	
	CC	39	13.5	8	6.1	
					
	T	370	64	190	72.5	**0.015**
	C	208	36	72	27.5	

Significant deviations (*p* < 0.05) are marked in bold.

**Table 2:** Interaction of rs4693608, rs4364254 and rs4426765 in CMV-seropositive and CMV-seronegative healthy individuals.

Group	Genotypes	CMV-Seropositive		CMV-Seronegative		Statistical Analysis
		№	Incidence (%)	№	Incidence (%)	
A	AA-TT-AA	48	17.7	29	24	**χ^2^ = 7.37** ***p* = 0.025**
	AA-TT-AC	10	3.7	7	5.8	
	AA-TC-AA	3	1.1	1	0.8	
	AA-TC-AC	16	5.9	5	4.1	
	AA-TC-CC	1	0.4	1	0.8	
	AA-CC-CC	1	0.4	0	0	
	AG-TT-AA	43	15.9	25	20.7	
	AG-TT-AC	2	0.7	0	0	
	AG-TC-AA	26	9.6	11	9.1	
		150	55.4	79	65.3	
B	**AG-TC-AC**	**43**	**15.9**	**13**	**10.7**	
	**AG-TC-CC**	**4**	**1.5**	**0**	**0**	
	**AG-CC-AC**	**10**	**3.7**	**1**	**0.8**	
	**AG-CC-CC**	**7**	**2.6**	**1**	**0.8**	
	**AG-CC-AA**	**2**	**0.7**	**0**	**0**	
		**66**	**24.4**	**15**	**12.4**	
C	GG-TT-AA	11	4.1	3	2.5	
	GG-TT-AC	1	0.4	1	0.8	
	GG-TC-AA	9	3.3	5	4.1	
	GG-TC-AC	20	7.4	12	9.9	
	GG-TC-CC	0	0	1	0.8	
	GG-CC-AA	1	0.4	1	0.8	
	GG-CC-AC	7	2.6	2	1.6	
	GG-CC-CC	6	2.2	2	1.6	
		55	20.3	27	22.3	

Significant deviations are marked in bold.

**Table 3:** Genotype and allele frequencies of the HPSE gene SNPs in CMV seropositive and seronegative AML patients.

SNP	Genotypes	CMV-Seropositive		CMV-Seronegative		*p*-Value
		№	Incidence (%)	№	Incidence (%)	
rs4693608	AA	45	26	10	43.5	0.29
	AG	92	53.2	8	34.8	
	GG	36	20.8	5	21.7	
					
	A	182	52.6	28	60.9	0.17
	G	164	47.4	18	39.1	
rs4693084	GG	103	62.4	16	69.6	0.55
	GT	55	33.3	7	30.4	
	TT	7	4.2	0	0	
					
	G	261	79.1	39	84.8	0.37
	T	69	20.9	7	15.2	
rs4426765	AA	82	49.1	16	72.7	0.078
	AC	70	41.9	6	27.3	
	CC	15	9	0	0	
					
	A	234	70.1	38	86.4	**0.024**
	C	100	**29.9**	6	**13.6**	
rs28649799	AA	129	77.2	19	86.4	0.61
	AG	37	22.2	3	13.6	
	GG	1	0.6	0	0	
					
	A	295	88.3	41	93.2	0.34
	G	39	11.7	3	6.8	
rs4364254	TT	62	**35.8**	17	**73.9**	**0.002**
	TC	91	**52.6**	4	**17.4**	
	CC	20	11.6	2	8.7	
					
	T	215	62.1	38	82.6	**0.006**
	C	131	**37.9**	8	**17.4**	

Significant deviations (*p* < 0.05) are marked in bold.

**Table 4:** Genotype and allele frequencies of the HPSE gene SNPs in CMV seropositive patients with hematological malignancies.

SNP	Genotype and Alleles	ALL		MDS		MM		NHL		*p*-Value to CMV-Seropositive Controls	*p*-Value to CMV-Seronegative Controls
		№	Incidence (%)	№	Incidence (%)	№	Incidence (%)	№	Incidence (%)		
rs4693608	AA	11	22	22	39.3	9	30	17	30.4	ALL: 0.66; 0.64	ALL: 0.12; 0.27
	AG	28	56	22	39.3	17	56.7	27	48.2	MDS: 0.2; 0.22	MDS: 0.91; 0.65
	GG	11	22	12	21.4	4	13.3	12	21.4	MM: 0.51; 0.39	MM: 0.24; 0.78
										NHL: 0.92; 0.71	NHL: 0.62; 0.73
	A	50	50	66	58.9	35	58.3	61	54.5		
	G	50	50	46	41.1	25	41.7	51	45.5		
rs4693084	GG	28	58.3	41	75.9	16	59.3	35	64.8	ALL: 0.25; 0.21	ALL: 0.36; 0.26
	GT	16	33.3	10	18.5	9	33.3	15	27.8	MDS: 0.12; 0.27	MDS: 0.16; 0.3
	TT	4	8.3	3	5.6	2	7.4	4	7.4	MM: 0.54; 0.41	MM: 0.6; 0.44
										NHL: 0.34; 0.65	NHL: 0.45; 0.69
	G	72	75	92	85.2	41	75.9	85	78.7		
	T	24	25	16	14.8	13	24.1	23	21.3		
rs4426765	AA	23	46.9	38	70.4	21	72.4	31	56.4	ALL: **0.034**; 0.086	ALL: **0.008**; **0.007**
	AC	17	34.7	10	18.5	7	24.1	20	36.4	MDS: **0.009**; 0.14	MDS: **0.034**; 0.79
	CC	9	**18.4**	6	**11.1**	1	3.4	4	7.3	MM: 0.12; 0.054	MM: 0.51; 0.3
										NHL: 0.85; 0.71	NHL: 0.65; 0.43
	A	63	64.3	86	79.6	49	84.5	82	74.5		
	C	35	**35.7**	22	20.4	9	15.5	28	25.5		
rs28649799	AA	42	85.7	49	89.1	23	79.3	50	89.3	ALL: 0.27; 0.75	ALL: 0.41; 0.75
	AG	6	12.2	6	10.9	4	13.8	5	8.9	MDS: 0.43; 0.21	MDS: 0.26; 0.21
	GG	1	2	0	0	2	6.9	1	1.8	MM: **0.003**; 0.26	MM: 0.09; 0.26
										NHL: 0.14; 0.32	NHL: 0.16; 0.32
	A	90	91.8	104	94.5	50	86.2	105	93.75		
	G	8	8.2	6	5.5	8	13.8	7	6.25		
rs4364254	TT	20	40	33	58.9	19	**65.5**	30	52.6	ALL: 0.89; 0.7	ALL: 0.084; 0.052
	TC	22	44	19	33.9	6	20.7	21	36.8	MDS: **0.049**; **0.015**	MDS: 0.53; 0.5
	CC	8	16	4	**7.1**	4	13.8	6	10.5	MM: **0.029**; 0.071	MM: 0.056; 0.6
										NHL: 0.3; 0.15	NHL: 0.5; 0.77
	T	62	62	85	75.9	44	75.9	81	71.1		
	C	38	38	27	24.1	14	24.1	33	28.9		

Significant deviations (*p* < 0.05) are marked in bold. The first *p*-value represents a correlation between genotypes and the second *p*-value marks a correlation between alleles.

**Table 5:** Univariate analysis of cumulative incidence of acute GVHD in association with patient enhancer and insulator HPSE gene SNPs and CMV infection.

Group of Analysis	Genotype or Status	Cumulative Incidence, (95%CI),%	χ^2^, *p*-Value
Patient CMV status	positive	**39.7(35.3–44.7)**	**4.7**
	negative	**24.1(15.0–38.7)**	**0.03**
Donor CMV status	positive	37.8(32.6–43.8)	0.49
	negative	40.7(33.2–49.9)	0.48
Patient-Donor Pairs	positive-positive	39.6(33.7–46.5)	
	positive-negative	42.7(33.9–53.8)	6.4
	negative-positive	24.0(11.9–48.2)	0.095
	negative-negative	19.1(7.9–46.0)	
Seropositive CMV patients	rs4693608:		
	AA	**49.6(41.2–59.7)**	**9.4**
	AG	**39.7(33.5–47.0)**	**0.0091**
	GG	**25.6(17.7–37.0)**	
	enhancer-insulator:		
	N-HR	**51.8(44.2–60.6)**	**16.9**
	N-MR	**36.7(30.1–44.7)**	**0.00022**
	N-LR	**22.5(15.0–33.8)**	
	Discrepancy:		
	D1	**58.9(47.4–73.3)**	**15.6**
	D2	**41.1(35.0–48.3)**	**0.00042**
	D3	**22.5(15.0–33.8)**	
Seronegative CMV patients	rs4693608:		
	AA	23.1(8.6–62.3)	0.16
	AG	22.2(11.0–45.0)	0.92
	GG	27.3(10.4–71.6)	
	enhancer-insulator:		
	N-HR	16.7(5.3–46.8)	3.5
	N-MR	34.8(19.9–60.9)	0.17
	N-LR	10.0(1.6–64.2)	
	Discrepancy:		
	D1	28.6(8.9–92.2)	1.3
	D2	25.8(14.2–46.8)	0.52
	D3	10.0(1.6–64.2)	
Seropositive-seropositive patient-donor CMV pairs	rs4693608:		
	AA	**52.3(41.5–66.0)**	**6.38**
	AG	**36.5(28.4–46.8)**	**0.04**
	GG	**27.3(16.8–44.2)**	
	enhancer-insulator:		
	N-HR	**51.3(41.4–63.5)**	**10.3**
	N-MR	**36.0(27.3–47.4)**	**0.0057**
	N-LR	**20.9(11.7–37.4)**	
	Discrepancy:		
	D1	**51.5(37.0–71.7)**	**6.9**
	D2	**41.1(32.9–51.3)**	**0.032**
	D3	**20.9(11.7–37.4)**	
Seropositive-seronegative patient-donor CMV pairs	rs4693608:		
	AA	47.8(31.2–73.3)	2.03
	AG	46.8(34.5–63.5)	0.36
	GG	28.6(14.5–56.2)	
	enhancer-insulator:		
	N-HR	53.3(38.2–74.5)	2.58
	N-MR	46.0(32.4–65.2)	0.28
	N-LR	26.1(13.1–51.9)	
	Discrepancy:		
	D1	58.3(36.2–94.1)	2.93
	D2	48.9(36.5–65.5)	0.23
	D3	26.1(13.1–51.9)	
Seronegative-seronegative and seronegative-seropositive patient-donor CMV pairs	rs4693608:		
	AA	27.3(10.4–71.6)	1.67
	AG	13.0(4.5–37.5)	0.43
	GG	30.0(11.6–77.3)	
	enhancer-insulator:		
	N-HR	18.8(9.4–41.7)	1.02
	N-MR	26.3(12.4–55.8)	0.6
	N-LR	11.1(1.8–70.5)	
	Discrepancy:		
	D1	28.6(8.9–92.2)	0.74
	D2	19.2(8.8–42.3)	0.69
	D3	11.1(1.8–70.5)	

Enhancer (rs4693608)—insulator (rs4426765 and rs4364254) SNP groups. N-HR: AA-AA-TT, AA-AA-TC, AA-AC-TT, AA-AC-TC, AA-NN-NN and AG-AA-TC genotype combinations (NN is any SNP genotype of rs4426765 and rs4364254 SNPs). N-MR: AG-AA-TT, AG-AC-TT, AG-AC-TC, AG-CC-TC, and GG-AA-TC genotype combinations. N-LR: GG-AA-TT, GG-AC-TT, GG-AC-TC, GG-AA-CC, GG-AC-CC, GG-CC-CC and AG-NN-CC genotype combinations (NN is any SNP genotype of rs4426765). Discrepancy between recipient and donor groups. D1: N-HR/N-MR, N-HR/N-LR; D2: N-HR/N-HR, N-MR/N-MR, N-MR/N-HR, N-MR/N-LR; D3: N-LR/N-LR, N-LR/N-MR, N-LR/N-HR. Significant deviations (*p* ≤ 0.05) and significant parameters for risk of acute GVHD are marked in bold.

**Table 6:** Association between enhancer rs4693608 and insulator rs28649799 and the number of CD34^+^ cells following administration of G-CSF to healthy donors after the first apheresis.

SNPs	Parameters	CMV-Seropositive				CMV-Seronegative			
		Genotype	Median (Range)	Comparisons to Carriers	*p*-Value	Genotype	Median (Range)	Comparisons to Carriers	*p*-Value
rs4693608	CD34^+^ × 10^6^ total yield	AA (48)	828.9	AA to GG	**0.013**	AA (25)	753.9	AA to GG	0.55
			(718.2–930.0)				(518.0–1008.0)		
		AG (72)	711.4	AA to others	**0.022**	AG (20)	878	AA to others	0.73
			(623.7–784.7)				(634.0–1062.0)		
		GG (38)	629.2	GG to others	**0.052**	GG (12)	781.3	GG to others	0.39
			(502.5–729.4)				(365.8–1184.0)		
	% CD34^+^	AA (47)	0.9	AA to GG	**0.022**	AA (25)	0.86	AA to GG	0.88
			(0.77–1.02)				(0.65–1.12)		
		AG (72)	0.73	AA to others	**0.007**	AG (20)	1.05	AA to others	0.34
			(0.7–0.81)				(0.85–1.34)		
		GG (37)	0.74	GG to others	0.23	GG (12)	0.82	GG to others	0.5
			(0.6–0.85)				(0.65–1.18)		
rs28649799	CD34^+^ × 10^6^ total yield	AA (121)	735.7	AA to AG	**0.089**	AA (39)	786.5	AA to AG	0.3
			(659.7–784.7)				(542.8–882.8)		
		AG (21)	854			AG (10)	1020.7		
			(648.0–1143.1)				(368.9–1485.7)		
	% CD34^+^	AA (120)	0.76	AA to AG	**0.031**	AA (39)	0.88	AA to AG	0.32
			(0.72–0.83)				(0.66–1.12)		
		AG (21)	0.97			AG (10)	1		
			(0.7–1.14)				(0.57–1.7)		

Significant deviations (*p* < 0.05) and trend to significance are marked in bold.

**Table 7:** Association analysis between combination of rs28649799 and rs4693608 SNPs and the number of CD34^+^ cells following administration of G-CSF to healthy donors after the first apheresis.

Parameters	Total				CMV-Seropositive				CMV-Seronegative			
	Genotype	Median (Range)	Comparisons to Carriers	*p*-Value	Genotype	Median (Range)	Comparisons to Carriers	*p*-Value	Genotype	Median (Range)	Comparisons to Carriers	*p*-Value
CD34^+^ × 10^6^ total yield	AA-AA (68)	786.6 (678.3–874.4)	AA-AA to AA-GG	0.074	AA-AA (45)	812.5 (718.2–930.0)	AA-AA to AA-GG	**0.032**	AA-AA (20)	733.5 (517.6–1129.2)	AA-AA to AA-GG	0.39
											
	AA-AG (68)	723.5 (634.0–789.2)	AA-AA to others	0.17	AA-AG (52)	701.6 (549.4–774.9)	AA-AA to others	**0.021**	AA-AG (12)	836 (545.9–1022.8)	AA-AA to others	0.97
											
	AA-GG (34)	643.9 (492.3–772.9)	AA-GG to others	0.086	AA-GG (24)	561.9 (466.1–729.4)	AA-GG to others	0.091	AA-GG (7)	772.9 (329.7–1196.6)	AA-GG to others	0.37
%CD34^+^	AA-AA (67)	0.88 (0.77–0.99)	AA-AA to AA-GG	**0.029**	AA-AA (44)	0.89 (0.76–1.02)	AA-AA to AA-GG	**0.021**	AA-AA (20)	0.86 (0.65–1.16)	AA-AA to AA-GG	0.41
											
	AA-AG (68)	0.75 (0.69–0.81)	AA-AA to others	**0.013**	AA-AG (52)	0.73 (0.6–0.77)	AA-AA to others	**0.003**	AA-AG (12)	1.11 (0.62–1.38)	AA-AA to others	0.71
											
	AA-GG (34)	0.74 (0.58–0.84)	AA-GG to others	0.15	AA-GG (24)	0.69 (0.45–0.83)	AA-GG to others	0.18	AA-GG (7)	0.74 (0.27–1.29)	AA-GG to others	0.23

Significant deviations (*p* < 0.05) are marked in bold.

## Data Availability

Not applicable.
